# Antibody for Serine 65 Phosphorylated Ubiquitin Identifies PLK1-Mediated Phosphorylation of Mitotic Proteins and APC1

**DOI:** 10.3390/molecules27154867

**Published:** 2022-07-29

**Authors:** Guy Mann, Prasad Sulkshane, Pradeep Sadhu, Tamar Ziv, Michael H. Glickman, Ashraf Brik

**Affiliations:** 1 Schulich Faculty of Chemistry, Technion–Israel Institute of Technology, Haifa 32000, Israel; guym@campus.technion.ac.il (G.M.); pradeepsadhu@campus.technion.ac.il (P.S.); 2 Faculty of Biology, Technion–Israel Institute of Technology, Haifa 32000, Israel; prasads@campus.technion.ac.il; 3 The Smoler Protein Research Center, Lorry I. Lokey Interdisciplinary Center for Life Sciences and Engineering, Technion Israel Institute of Technology, Haifa 32000, Israel; tamarz@technion.ac.il

**Keywords:** posttranslational modifications, antibody off-targets, cell cycle

## Abstract

Deciphering the protein posttranslational modification (PTM) code is one of the greatest biochemical challenges of our time. Phosphorylation and ubiquitylation are key PTMs that dictate protein function, recognition, sub-cellular localization, stability, turnover and fate. Hence, failures in their regulation leads to various disease. Chemical protein synthesis allows preparation of ubiquitinated and phosphorylated proteins to study their biochemical properties in great detail. However, monitoring these modifications in intact cells or in cell extracts mostly depends on antibodies, which often have off-target binding. Here, we report that the most widely used antibody for ubiquitin (Ub) phosphorylated at serine 65 (pUb) has significant off-targets that appear during mitosis. These off-targets are connected to polo-like kinase 1 (PLK1) mediated phosphorylation of cell cycle-related proteins and the anaphase promoting complex subunit 1 (APC1).

## 1. Introduction

Ubiquitination and phosphorylation are crucial protein posttranslational modifications (PTMs) involved in regulating myriad cellular processes. Often, both of these PTMs are involved as part of a “PTM code” to direct correct progression of biological pathways [1]. One such pathway is the removal of damaged mitochondria by a selective autophagy [2] process known as mitophagy [3]. During mitophagy, PTEN-induced kinase 1 (PINK1) is stabilized at mitochondrial damage sites and phosphorylates ubiquitin (Ub) at serine 65 to generate phosophoUb (pUb) [4]. PINK1-mediated phosphorylation of Ub conjugated to proteins at the mitochondrial outer membrane, such as TOM20 [5], facilitates polymerization of poly(p)Ub chains, which further recruit and activate the E3 ligase Parkin; thereby amplifying the poly(p)Ub signal to recruit autophagy components [6]. To the best of our knowledge, mitophagy is the only reported cellular process that specifically utilizes pUb, and PINK1 is the only reported kinase that is specific for phosphorylating Ub at serine 65 [7,8]. Importantly, mitophagy is crucial for maintaining the correct the energy regime in many cells including neurons [9]. As a result, failure to eliminate damaged, dysfunctional and obsolete mitochondria can lead to a number of pathophysiological conditions, including neurodegeneration [10]. In addition to their role during mitophagy, ubiquitination and phosphorylation have crucial roles during mitosis [11,12]. Consequently, several enzymes that install these modifications are considered “master regulators” of mitosis, including the ubiquitin (Ub) E3 ligase anaphase promoting complex (APC) [13] and polo-like kinase 1 (PLK1) [12].

Antibodies are a principle component of the adoptive immune system and are extremely useful tools for detecting proteins and their modified forms. Antibodies can be isolated from both mono- and polyclonal lymphocyte populations to generate monoclonal and polyclonal antibodies that are fundamentally different in their specificity and sensitivity [14]. As of today, modification-specific antibodies are the gold standard in imaging the localization, stability and binding partners of posttranslationally modified proteins in biological samples [15]. Unfortunately, detecting PTMs demands rigorous characterization of these antibodies to avoid off-targets, non-specific binding and binding to the unmodified protein [16,17]. This is particularly important when developing antibodies that must detect a PTM at a specific protein site [18], such as antibodies specific for phosphorylation at Serine 65 of ubiquitin (pUb) that is conjugated onto a substrate protein. Antibody off-targets are characteristic to a particular antibody; therefore, employing non-specific controls (i.e., isotype controls) does not solve this issue, which is crucial for many applications including immunoprecipitation (IP) and subsequent analysis of PTM binders using mass spectrometry [19]. Adding to the complexity, the off-targets of PTM-specific antibodies might only appear under conditions that induce a particular modification state. As a result, using antibodies for PTM studies requires careful evaluation of their specificity in various experimental conditions in order to avoid artifacts.

Site-specifically modified peptides and proteins are powerful tools for both generating PTM specific antibodies and to validate their specificity [20]. As of today, the most direct approach to prepare these is via chemical peptide and protein synthesis (CPS) [21]. Using CPS, various modified proteins were studied in vitro and promoted our understanding of their PTMs [22]. In particular, we have previously prepared a variety of ubiquitinated [23], phosphorylated [24] proteins and phosphorylated Ub and diUb [25] to shed light on the biochemical properties of these unique modifications. For example, using tetra-ubiquitinated synthetic proteins, we probed the Ub chain requirements for the 26S and 20S proteasome units in protein degradation [23,26,27].

Recent developments in protein delivery tools now enable the application of modified synthetic proteins to deliver and study them inside live cells [28,29,30]. Using these tools, we have successfully delivered a stable phosphorylated Ub probe to study its involvement during mitophagy [28]. Notably, our synthetic probe was strongly identified by commercially available pUb antibodies, which prevented us from simultaneously comparing the native pUb and our synthetic probe. Here, we demonstrate that the most cited pUb antibody (citeab database [31]) has significant off-target binding with cell cycle-related proteins during mitosis. These off-targets correlate with mitotic phosphorylation events and are strongly identified by the antibody for pUb. Moreover, we identify the subunit 1 of the Ub E3 ligase, anaphase promoting complex (APC), as one of these off-targets.

## 2. Results

**Figure 1:**
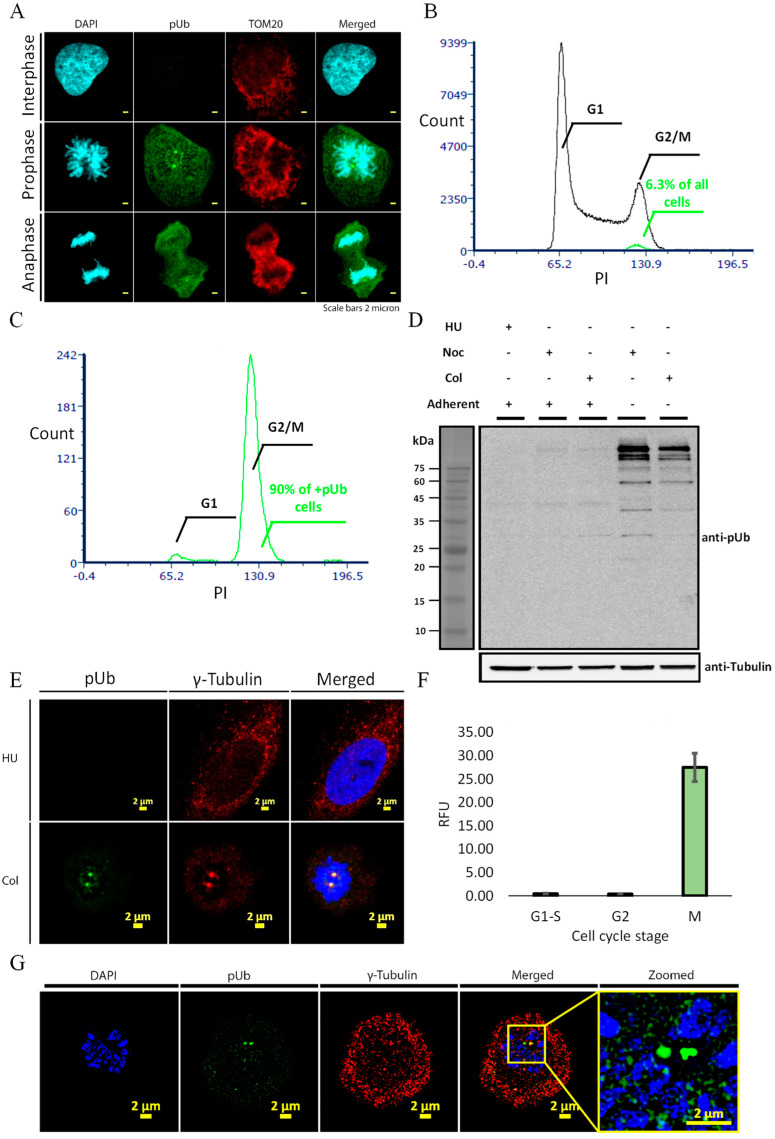
Antibody for pUb recognizes and binds mitosis-related phosphorylation events in U2OS cells. (**A**) Confocal imaging of interphase and mitotic U2OS cells with pUb (green) and TOM20 (red) antibodies and DAPI (cyan); (**B**) cell cycle distribution of unsynchronized U2OS cell stained with propidium iodide (PI) and AF488 conjugated pUb antibody. Green overlay is pUb positive cells; (**C**) histogram of PI intensity under pUb positive gate shows that almost all pUb positive cells are in G2/M stage; (**D**) Western blot analysis of U2OS cells in different cell cycle stages with pUb antibody. Representative result of three independent experiments; (**E**) confocal imaging of interphase and mitotic U2OS cells with pUb (green) and γ—Tubulin (red) antibodies and DAPI (blue); (**F**) quantification of pUb signal fluorescence intensity from cells in D using γ—Tubulin mask; (**G**) SIM of interphase and mitotic U2OS cells with pUb (green) and γ—Tubulin (red) antibodies and DAPI (blue). Scale bar for images are 2 micron. Flow cytometry histograms are representative results from three independent experiments (>10,000 cells each).

During our previous studies of synthetic Ub probes during mitophagy, we used a laser scanning confocal microscope (LSCM) to image ubiquitin phosphorylation during mitophagy in U2OS cells [28]. In these experiments, the cells were stained using antibodies for TOM20 and a polyclonal anti-pUb. Surprisingly, without inducing mitophagy, we observed a strong pUb signal that appeared at centrosomes specifically in cells containing condensed mitotic chromosomes (Figure 1A). Excited by this unexpected observation, we set out to study whether this pUb signal has any cell cycle specific involvement. We performed a flow cytometry analysis using an alexa flour 488 (AF488) conjugated anti-pUb antibody, to correlate between the pUb signal and cell cycle stage in unsynchronized (US) U2OS cells. Surprisingly, we observed that the pUb signal was only present in a fraction of the G2/M subpopulation, corresponding to the reported fraction (~6%) of mitotic U2OS cells (Figure 1B and Figure S1A,B) [32]. Furthermore, under a pUb gate, only cells with duplicated DNA were counted, confirming that pUb positive cells are in a subpopulation of G2/M cell cycle stage (Figure 1C).

When entering mitosis, U2OS cells lose their adherence and allow specific isolation of mitotic cells by “mitotic shake-off” from the adherent G2 subpopulation [32]. To confirm that the pUb signal is specific to mitotic cells, we synchronized U2OS cells to G1/S stage using hydroxyurea (HU) and to G2/M stage using nocodazole (Noc) or colcemid (Col) (Figure S1C) [32]. By combining mitotic shake-off with cell cycle inhibitors, we were able to isolate a well-defined non-adherent mitotic population and adherent G2 population from the same samples. In these populations, Col treatment significantly increased the overall percent of in G2/M cells expressing pUb (Figure S1D) with an additional increase upon separating the G2 and M cells by mitotic shake-off (Figure S1E). Western blot analysis of synchronized cells confirmed that this antibody strongly recognizes several high molecular weight bands that are highly specific to mitotic cells (Figure 1D). Importantly, the pUb bands were not visible in the adherent Noc and Col treated cells, confirming that pUb does not increase in the G2 cell population.

We then examined the subcellular distribution of the mitotic pUb signal by LSCM and structure illumination microscopy (SIM) of synchronized cells immunostained with pUb and γ-Tubulin (as a centrosome marker) antibodies. Our results suggested that the pUb signal is present at centrosomes, kinetochores and in cytoplasm, but is excluded from the condensed chromosomes (Figure 1E,G). Image analysis using γ-Tubulin masking confirmed that pUb intensity in centrosomes increases in mitotic (M) phase cells (Figure 1F).

**Figure 2:**
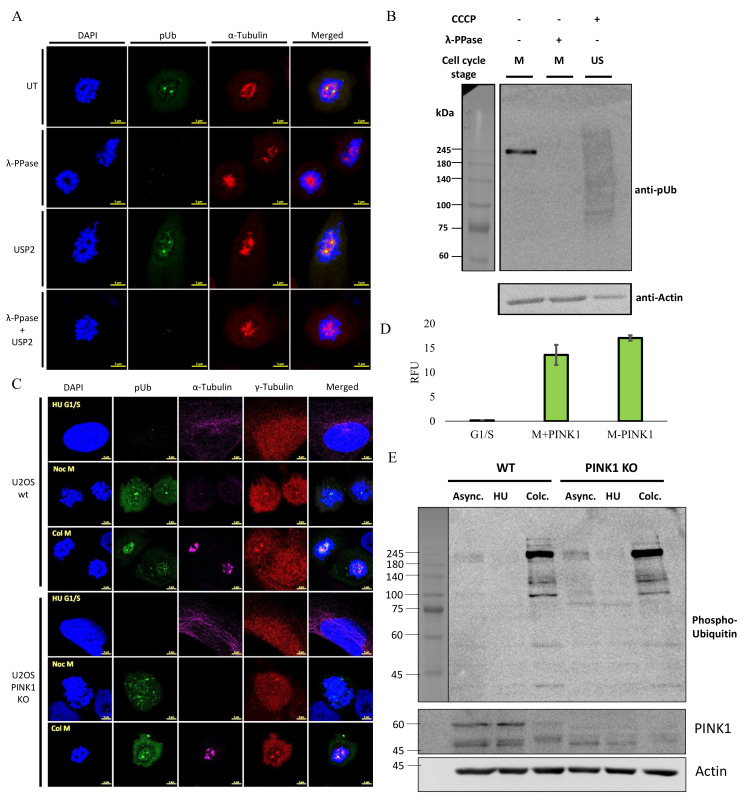
Signal from pUb during mitosis is independent of PINK1 and is abolished by λ—PPase. (**A**) Confocal images of mitotic cells treated with λ—PPase, USP2 and both enzymes and stained with pUb (green), α—Tubulin (red) antibodies and DAPI (blue); (**B**) Western blot analysis using pUb antibody of mitotic lysate with and without λ—PPase treatment. A positive control for presence of pUb are extracts of unsynchronized U2OS cells treated with CCCP; (**C**) confocal images of wt and PINK1 KO U2OS cells stained with antibodies for pUb (green), α—Tubulin (magenta), γ—Tubulin (red) and DAPI (blue) in different cell cycle stages; (**D**) quantification of pUb signal in C (γ—Tubulin mask) from two independent experiments (>100 cells each); (**E**) Western blot analysis using pUb antibody of WT and PINK1 KO U2OS cells in different cell cycle stages. Representative image of two independent experiments. Western blot signals are HRP luminescence. Scale bars are 5 micron.

Studying Ub phosphorylation at serine 65, as a second PTM layer, introduces additional complications and requires rigorous confirmation that both PTMs are indeed found on the same substrate at the same site. We therefore turned to confirm that the mitotic pUb signal depends simultaneously on both Ub and phosphorylation. For this, we compared the pUb signal in fixed U2OS cells that were incubated with promiscuous enzymes; lambda phosphatase (λ-PPase) and the general deubiquitinase (DUB) ubiquitin specific protease 2 (USP2). Treating mitotic cells with λ-PPase completely abolished the pUb signal in these cells, confirming that the identified mitotic pUb labeling is phosphorylation-dependent (Figure 2A). However, the USP2 treatment did not affect the pUb signal (Figure 2A). USP2 catalyzes the removal of Ub from its substrates, but cannot eliminate the phosphorylation signal; we therefore decided to further confirm the resistance of this pUb signal to the action of DUBs by Western blot using anti-pUb. It is widely reported that phosphorylation of polyUb chains at S65 renders them more stable to DUBs [25]. We therefore treated mitotic lysates with a panel of DUBs that were reported to hydrolyze pUb chains [33] and USP2. Treating mitotic lysates with these DUBs did not have a significant effect on the pUb signal, suggesting the pUb signal in this context is not removable by DUBs (Figure S2). In addition, we incubated mitotic lysates with λ-PPase and compared the pUb signal to both untreated mitotic lysate and unsynchronized parkin expressing U2OS cells, treated with the mitochondrial damaging agent carbonyl cyanide 3-chlorophenylhydrazone (CCCP) [28] as a positive control for phosphorylated ubiquitin. In our hands, and despite similar sample preparation, the pUb signal that arose in mitosis differed fundamentally from the CCCP-induced pUb signal (Figure 2B). In contrast to the characteristic pUb “smear” resulting from mitophagy induction upon CCCP treatment, the pUb signal specific to mitotic phase cells was more intense and visible as a few discrete bands, suggesting that a limited number of proteins contain this modification (Figure 2B).

As of today, PINK1 is the most characterized Ub kinase and is the only known kinase to specifically phosphorylate Ub at serine 65 [3]. To examine PINK1’s involvement in the mitotic pUb signal, we used the CRISPR/Cas9 approach to knock-out PINK1 in U2OS cells with and without a stable expression of untagged human Parkin [28,34] (Figure S3A). Surprisingly, knocking out PINK1 did not affect the specific mitotic phase pUb signal, suggesting that this signal is distinct from the mitophagy-related Ub phosphorylation by PINK1 (Figure 2C–E). Furthermore, Parkin expression had no significant effect on the mitotic pUb signal (Figure S3B). Despite the fact that the mitotic pUb signal is independent of PINK1, PINK1 knockout cells showed significantly slower proliferation (Figure S4), which is in agreement with its role in progression of the cell cycle [35].

**Figure 3:**
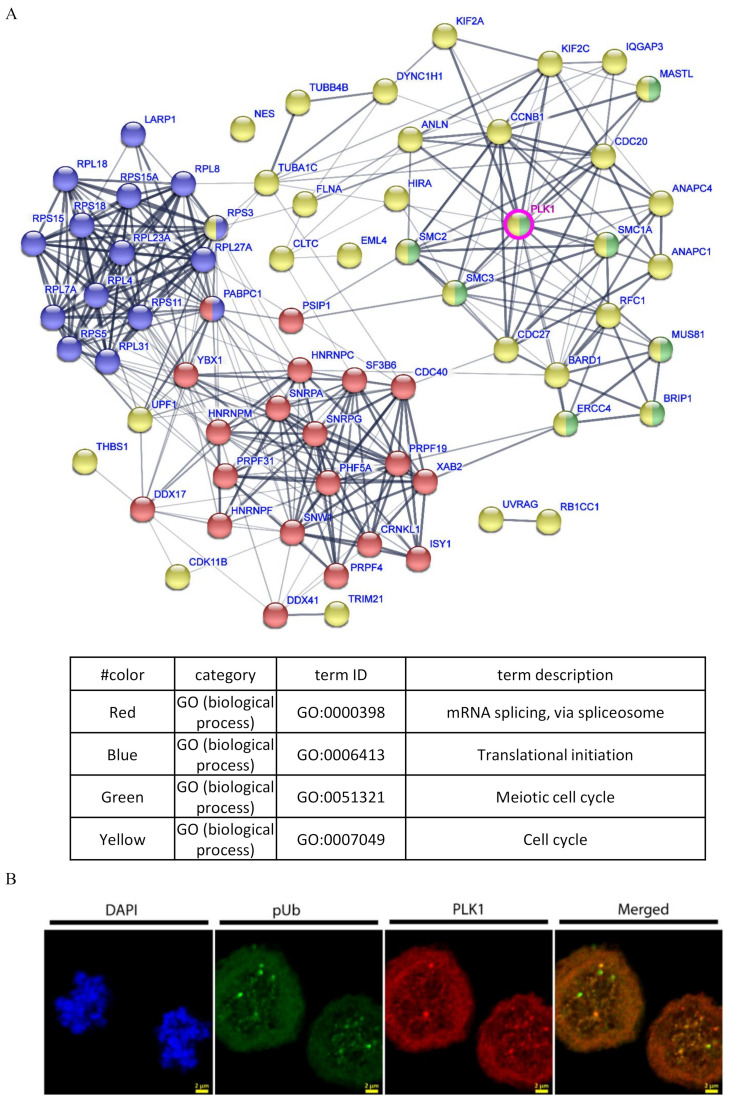
Immunoprecipitation from mitotic cells using the pUb antibody enriches various cell cycle-related proteins and the master regulator PLK1. (**A**) STRING analysis of proteins pulled down using anti-pUb from mitotic cells (Isotype control is anti-HA antibody). Only proteins that were significantly enriched by this antibody in three independent experiments are shown. Cell cycle, mitosis and mRNA splicing are highlighted in red, blue and green, respectively. PLK1 is highlighted by pink circle; (**B**) confocal image of mitotic U2OS cells stained for pUb (green), PLK1 (red) and DAPI (blue) show similar distribution in centrosomes. Scale bars are 2 micron.

The distinct gel migration of mitotic proteins immunoblotted using pUb convinced us that this signal might be specific to a certain protein or a set of proteins that migrates to the same extent on SDS-PAGE. Therefore, we aimed to enrich and identify the proteins in these bands for subsequent analysis using proteomics. First, using lysates from both G1/S and M phase cells, we confirmed that this antibody is compatible with immunoprecipitation (IP) for enriching mitosis-related pUb-conjugated proteins (Figure S5A). To identify the proteins that are conjugated or associate with the pUb signal, we performed IP using the pUb antibody from mitotic lysates alongside with an isotype control antibody. The interacting proteins were eluted from the antibody and identified by liquid chromatography with tandem mass spectrometry (LC-MS/MS). No phosphorylation on ubiquitin was identified. Quantification of the intensities of those proteins compared to the isotype control was based on peak areas of the identified peptides. Proteins that were significantly enriched (>2 fold, Table S1, supplementary file) were analyzed for annotation enrichment (using the STRING tool) [36] to reveal a prominent cluster of cell cycle-related proteins with PLK1 at its center (Figure 3A). We further observed that PLK1 and pUb show similar distribution to centrosomes during mitosis (Figure 3B).

We then focused on proteins that have a similar molecular weight to the observed pUb bands, including PRPF8, BAZ2A, SNRNP200, RIF-1, CENP-F, 53BP1 and APC1. Western blot analysis of mitotic lysates, enriched by pUb antibody, revealed that APC subunit 1 (APC1) is the only examined protein that overlaps with the pUb bands (Figure S5B). As centrosome protein F (CENPF) was an abundant protein in our pUb IP from mitotic lysates; we suspected that CENPF might be covalently conjugated with pUb. Therefore, we performed immunoprecipitation of CENPF, but failed to detect pUb bands in the IP fractions, suggesting that CENPF may not be covalently modified by pUb (Figure S6).

**Figure 4:**
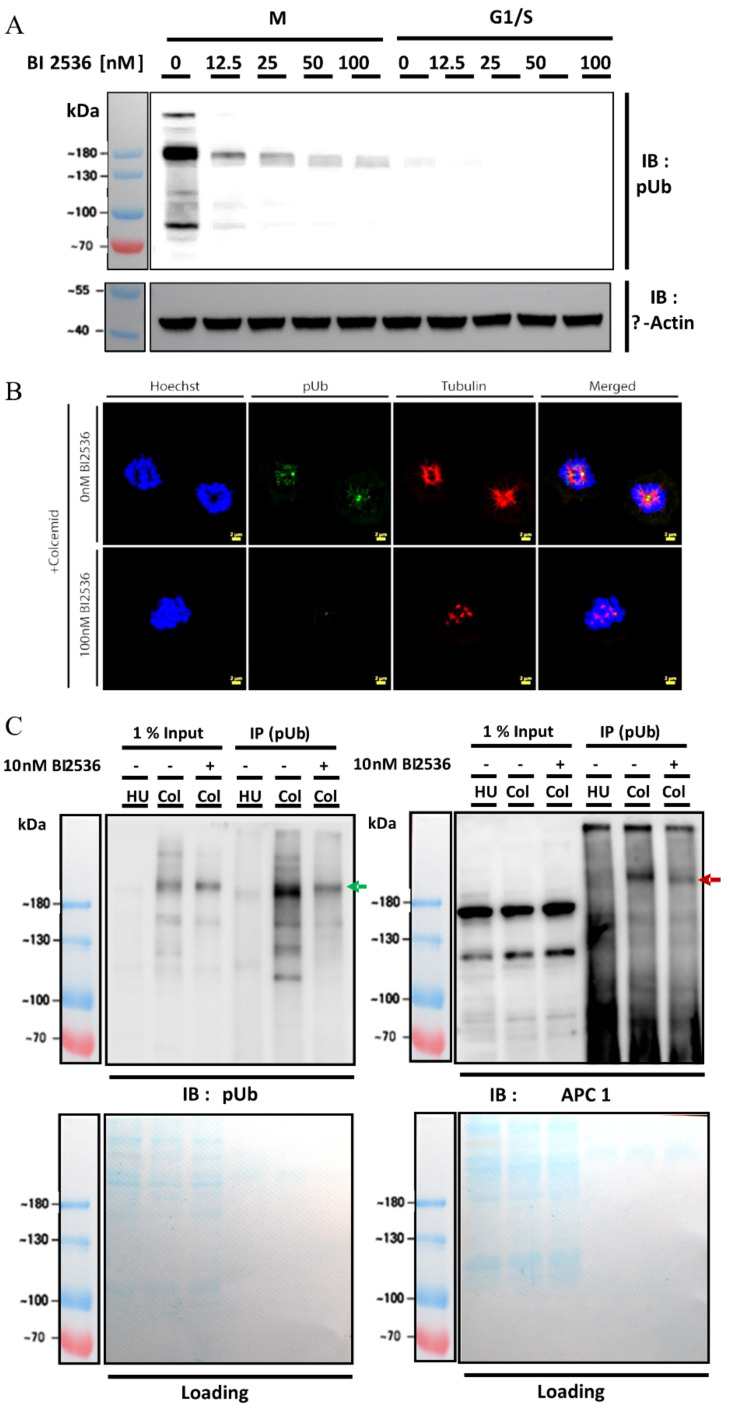
Mitotic pUb signal is PLK1 dependent and correlates with APC1. (**A**) Western blot analysis of mitotic cells treated with and without PLK1 inhibition; (**B**) confocal image of mitotic cells with and without PLK1 inhibition; (**C**) APC1 is enriched in mitotic cells by pUb antibody and PLK1 inhibition reduces the APC1 enrichment by pUb antibody from parallel electroporation and Western blot analysis of same samples using different antibodies. Arrows mark the similarly migrating band detected by both antibodies. Representative result of two independent experiments. Loading control is InstaBlue^TM^.

Since PLK1 is a central kinase during mitosis and it was also detected in the IP fractions of pUb from mitotic lysates, we examined the involvement of PLK1′s involvement in generating the mitotic pUb signal by inhibiting PLK1 in U2OS synchronized to G1/S and mitosis cell cycle stages. Treating cells with the PLK1 inhibitor BI2536 [37] followed by analyzing the pUb signal by Western blot, confocal imaging and flow cytometry revealed that the mitotic pUb signal was abolished (Figure 4A,B and Figure S7A). This effect of PLK1 inhibition suggests that PLK1 is possibly involved in generating the pUb signal. Nevertheless, we cannot exclude that PLK1 inhibition indirectly abolishes the pUb signal due to the central regulatory function of PLK1 during mitosis.

Next, we examined if the gel migration of the proteins identified by LC-MS/MS could be affected by PLK1 inhibition. For this, we again used antibodies for the most significantly enriched proteins in crude lysates from mitotic cells treated with and without BI2536 treatment. We analyzed several duplicates of the same mitotic lysates (with and without PLK1 inhibition) in the same gel and compared the migration of CENP-F, 53BP1, PRPF8, BAZ2A, APC1 and RIF1 to pUb (Figure S7C–F). Of the examined proteins, APC1 was most significantly affected by PLK1 inhibition (Figure S7F). Notably, we could not detect any bands that both overlap with the pUb bands and effected by PLK1 inhibition in these crude lysates.

In order to eliminate the possibility that the pUb signal does not affect the protein’s migration or represents a sub-stoichiometric population, we used the pUb antibody to enrich proteins from lysates in G1/S stages, mitosis, and mitotic cells that were treated with BI2536. Western blot using an antibody for APC1 revealed a similarly migrating band that was diminished by BI2536 treatment in both APC1 and pUb blots (Figure 4C). This suggests that APC1 is recognized by pUb antibody as a result of PLK1-mediated phosphorylation during mitosis. Notably, this modification was not identified by APC1 antibody in the inputs due to its sub-stoichiometric nature.

**Figure 5:**
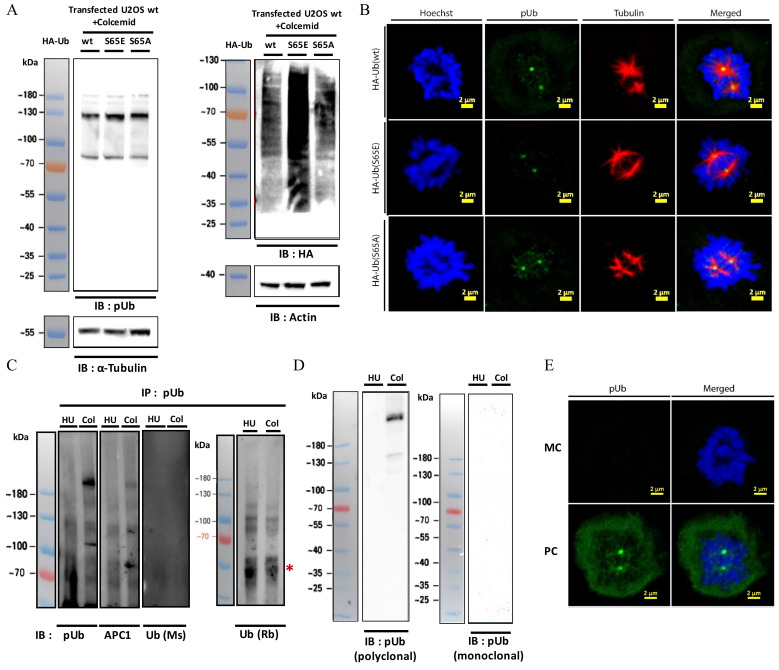
Overexpression of Ub S65 mutants does not affect pUb signal in mitosis. (**A**) Western blot analysis of mitotic cells transfected with HA tagged Ub mutants using pUb and HA antibodies; (**B**) confocal images of mitotic cells transfected with the relevant Ub mutants stained with pUb and α-Tubulin antibodies. Scale bars are 2 micron; (**C**) mitotic lysates of U2OS cells were subjected to immunoprecipitation with pUb antibody and the immunoprecipitates were probed with pUb, APC1 and Ub antibodies; (**D**) Western blot analysis of mitotic cell lysates using monoclonal (MC) and polyclonal (PC) pUb antibodies; (**E**) confocal images of mitotic cells stained with MC and PC pUb antibodies. Western blot signals are HRP chemiluminescence and are shown as false fluorescence. Scale bars are 2 micron. * antibody heavy chain.

After confirming that APC1 is identified in mitosis by pUb antibody, we examined if APC1 is indeed ubiquitinated prior to its phosphorylation. For this, we transfected U2OS cells with plasmids encoding human influenza hemagglutinin (HA) tagged wild type ubiquitin and its S65A and S65E mutants to evaluate if the pUb signal diminish upon overexpression of the Ub mutants (Figure S8A). Surprisingly, ectopic expression of these proteins did not affect the pUb signal in Western blot, confocal imaging (Figure 5A,B) and flow cytometry (Figure S8B). Alternatively, we examined the presence of Ub in the pUb band using two commercial antibodies for Ub produced in mouse and rabbit. Using these antibodies, we did not identify any specific bands in the mitotic lysates that were enriched by pUb antibody (Figure 5C). Furthermore, we used bead loading [38] to deliver a previously reported TAMRA labeled synthetic Ub into live U2OS cells [29]. Immunofluorescence using a pUb AF488-conjugated antibody revealed that TAMRA-Ub’s cellular distribution in mitotic cells is significantly different then pUb’s (Figure S9).

In these experiments, we could not obtain additional evidence that the mitotic pUb signal is related to Ub. Consequently, we suspected that the pUb antibody identifies mitotic phosphorylation events that are unrelated to serine 65 in Ub, despite the wide application of this pUb antibody in numerus studies. To the best of our knowledge, there are only two commercially available pUb antibodies that are compatible with detecting endogenous pUb. Therefore, we compared the polyclonal anti-pUb used in the current study with a mouse monoclonal anti-pUb (MC-anti-pUb). Comparison between these antibodies by Western blot and CLSM revealed that MC-anti-pUb does not recognize the previously observed mitotic specific pUb signal (Figure 5D,E).

As an alternative approach to detect pUb that is independent of antibodies, phosphoproteome of US and mitotic cell tryptic were compared. Peptides from the different lysates were labeled with heavy or light isotopes of formaldehyde using reductive dimethylation. The labeled peptides were combined and enriched the phosphopeptides using TiOx2 beads followed by for LC-MS/MS analysis. Unfortunately, in these experiments, we could not detect any increase in the intensity of pUb peptides in mitotic cells (result not shown). To validate our protocol, we included a positive control of US Parkin overexpressing U2OS cells that were treated with CCCP for four hours. In this experiment, pUb peptide was detected in all conditions, with the CCCP treated cells providing over 130 times stronger pUb peptide intensity compared to the mitotic cells (Figure S10).

## 3. Discussion

As of today, detecting, enriching and imaging post-translationally modified proteins still depends on the repertoire of available site-specific antibodies [18]. Despite the rigorous procedures employed to confirm that they are indeed site-specific, this characterization cannot cover the full spectrum of polypeptides that can exist within the ever-changing environment inside living cells. Furthermore, subpopulations in the same culture can undergo different metabolic adaptations that are disproportionally represented in the overall population. Therefore, even the most characterized antibodies can have unknown off-targets that complicates PTM studies.

Our results suggest that a widely used commercially available polyclonal pUb antibody strongly identifies mitotic phosphorylation events in addition to its original pUb target. The high affinity binding of this antibody to the centrosomes/spindle poles and the key role of PINK1 during mitotic progression compelled us to investigate the pUb involvement during mitosis, which is an intense hub of diverse PTM’s, dominated by phosphorylation and ubiquitination. We found that these phosphorylations are introduced by PLK1 during mitosis and specifically found on the APC subunit 1. The complexity in studying mitotic PTMs prevented us from completely excluding that “real” pUb modification is involved in cell cycle, but we suggest that the identified phosphorylation is not directly related to Ub or occurs in parallel to ubiquitination, at a different site. Despite the strong pUb signal obtained by this specific antibody in multiple assays, we were unable to detect the pUb peptide in mitotic cell lysates by mass spectrometry.

APC has crucial involvement in regulating the cell cycle, is comprised of a large complex and is tightly regulated by complex PTM’s. Therefore, the off-targets of this antibody could assist in examining the position and function of the identified APC1 phosphorylation(s) during mitosis. In principle, one can also examine antibody specificity by comparing the cellular distribution of fluorescently tagged synthetic proteins, with and without PTMs, to the commercially available site-specific PTMs antibodies in live cells through multiplexed protein delivery [29].

We urge others to exercise caution when using this particular pUb antibody, specifically for pull-down and proteomic analysis, to avoid undesired enrichment of phosphorylated mitotic proteins. Furthermore, disposing of mitotic populations is important before using this antibody to examine PINK1′s enzymatic activity or mitophagy progression.

## 4. Materials and Methods

### 4.1. List of Tissue Culture Reagents

U2OS (HTB-96™) were purchased from ATCC^®^. U2OS cells with stable expression of untagged human Parkin were produced as previously reported [28,29]. Dulbecco’s modified eagle’s medium low glucose (DMEM-LG), phenol free DMEM (Opti-DMEM), fetal bovine serum (FBS), L-Glu, antibiotics (penicillin/streptomycin), trypsin/EDTA and phosphate buffered saline (PBS) were purchased from biological industries. Hoechst 33342 solution (20 mM), Mitotracker green (L7526), deep red (M22426) and Lysotracker blue (L7525) probes were purchased from Thermo-Fisher. The 8-well µ-Slide for live cell and 8-well chambered removable immunofluorescence slides for confocal microscopy were purchased from ibidi, and 2-((3-chlorophenyl)hydrazono)malononitrile (CCCP) and poly-L-lysine hydro bromide was purchased from sigma.

### 4.2. Cell Culture

U2OS cells were cultured in DMEM-LG (low glucose) supplemented with 10% FBS, 0.2 mM L-Glu and antibiotics (penicillin/streptomycin) in a humidified 37 °C incubator at 5% CO_2_. To detach cells from culture flasks, the media was aspirated and the flask was washed with sterile calcium and magnesium free PBS before cells were treated with 0.25%

Trypsin + 0.02% EDTA solution and returned to the incubation chamber for 5 min. Trypsin was quenched by adding the FBS supplemented media. The cell suspension was collected and the cells were pelleted (2 min at 1000× *g*). Media was then aspirated and the cell pellet was re-suspended in fresh media. The cell density was determined using a Countess II automated cells counter. For confocal microscopy, cells were seeded on poly-L-lysine (PLL)-treated 8-well chamber slides (Ibidi) with a removable silicone chamber (cat # 80841) in 3 × 10^4^ cells/well and were allowed to reach ~90% confluence (24 h).

### 4.3. Enrichment of Cells in G1/S Cell Cycle Stages

U2OS cells were grown on 150 mm plates to reach 70% confluence. A day before the experiment, cells were treated with full culture medium containing hydroxyurea (Tzamal D-chem, A10831, 4 mM) and incubated for 12–24 h. Cells were harvested by trypsinization prior to lysis. Alternatively, we enriched cells to G1/G0 stages by culturing in DMEM containing 0.5% serum (serum starvation) for 48 h (Figure S10).

### 4.4. Enrichment of Cells in G2 and Mitosis Cell Cycle Stages

U2OS cells were grown on 150mm plates to reach 70% confluence. Cells were treated with full culture medium containing colcemid (Sigma, D7385, 0.04 µg/mL) or nocodozole (Mercury, MBS487928, 0.5 µg/mL) and incubated for 12 h. Prior the each experiment, mitotic cells were harvested by mitotic shake-off [32] and adherent G2 cells were harvested by trypsinization. For PLK1 inhibition, cells were treated with a mixture of colcemid and BI2536 and harvested by mitotic shake-off after 12 h incubation.

In our imaging experiments, the mitotic cells were not separated by mitotic shake-off. Alternatively, mitotic cells were identified by the appearance of condensed chromosomes in the DAPI channel as a morphological indication of mitosis.

### 4.5. Plasmid Transfection

Cells were grown on 60 mm plates to reach 70% confluence. Four micrograms of plasmid DNA was mixed with lipofectamine 3000 reagent (using manufacturer recommended procedure, Invitrogen, Waltham, MA, USA). Briefly, the DNA-lipofectamine complexes in Opti-MEM medium were incubated for 15 min at room temperature and added dropwise to the cells and incubated for 16–24 h prior to the experiments.

### 4.6. Loading Cells with TAMRA-Ub

U2OS cells were cultured in PLL-treated removable chamber 8-well chamber slides (ibdi, 80841) to 70% confluence. A day before fixation, cells were interphase and mitotic cell cycle stages were enriched by incubation with hydroxyurea and colcemid, as described above. After 12 h of incubation with the cells cycle inhibitors, the previously reported TAMRA-Ub probe [29] was diluted from concentrated 1000X DMSO stocks into sterile PBS containing 0.1% pluronic^®^ F-68 (24040032, Gibco). The protein concentration was determined by Bradford assay against a BSA calibration curve and the concentrations were adjusted to a final of 6 μM. For loading, the culture medium was removed and cells were gently washed once with warm PBS + 0.1% pluronic^®^ F-68 solution. To the washed cells, 150 µL of the TAMRA-Ub solution was added (to cover the cells) and a monolayer of sterile glass beads (Sigma Aldrich< 106 μm, G4649) was sprinkled over the cells. The culture slide was tapped on a bench eight times, with gentle swirling of the slide for a total of two times in between tapping, and incubated for 2 min at 37 °C under 5% CO_2_, as described previously [29]. Following incubation, the cells were gently washed with DMEM (0.3 mL × 2) and incubated at 37 °C under 5% CO_2_ with colcemid containing DMEM for 4 h. Cells were fixed, stained using an AF488 conjugated pUb antibody and mounted using Fluoromount-G™ solution containing DAPI (Invitrogen, 00-4958-02).

### 4.7. Protein Extraction and Western Blotting

The mitotic cell population was collected by “mitotic tapping” and by vigorously flushing the weakly adherent cells with either culture media or by PBS. The adherent cells were harvested by trypsinization and pelleted by centrifugation at 500× *g* for 5 min. The cell pellets were washed twice with cold PBS and lysed with ice-cold NP-40 lysis buffer (50 mM Tris pH 7.4, 150 mM NaCl, 0.5% NP-40) containing protease and phosphatase inhibitor cocktails. The cell lysates were centrifuged at 14,000× *g* to obtain the soluble fraction. An equal amount of whole cell extracts were resolved by SDS-PAGE and electroblotted onto PVDF membranes followed by blocking in 5% skimmed milk (*w*/*v*) in TBST for 1 h at room temperature. The membranes were incubated with appropriate primary antibodies for either 1 h at room temperature or at 4 °C overnight followed by incubation with corresponding secondary antibodies. Following washing with TBST, the membranes were incubated with a mixture of luminol and H_2_O_2_ and the signals were captured using the ECL detection system.

### 4.8. Immunoprecipitation (IP)

For a typical coimmunoprecipitation reaction, at least 2 mg of the whole cell lysate (as obtained above) per reaction were first precleared by incubation with protein A/G-Agarose beads for 1 h at 4 °C on a tube rotator with gentle agitation. The beads were pelleted down by a spin at 500× *g* for 5 min at 4 °C. The precleared supernatant thus obtained was incubated with appropriate antibody for overnight at 4 °C with gentle agitation. Next day, this lysate was incubated with protein A/G-Agarose beads for 3 h at 4 °C with gentle agitation followed by a brief centrifugation (500× *g* for 5 min) to harvest the beads. The beads were washed at least 5 times with ice-cold cell lysis buffer. The beads were finally boiled in Laemmli sample buffer for about 3 min following which they were briefly spun and the supernatant was loaded onto the gel for further analysis.

### 4.9. Coimmunofluorescence

The cells were cultured in 8-well removable microchamber slides (ibidi, 80841) overnight (~90% coverage). Following the desired treatments, the culture media was carefully aspirated and the cells were gently washed with warm PBS at least 2 times before being fixed with warm 4% (*w*/*v* in PBS) paraformaldehyde (PFA) at room temperature for 20 min. The cells were then permeabilized with 1% (*v*/*v* in PBS) Triton X-100 for 5 min followed by blocking with 5% (*w*/*v* in PBS) BSA for 1 h at room temperature before being washed with PBS at least two times. The cells were then incubated with appropriately diluted primary antibodies either overnight at 4 °C or for 1 h at room temperature. Following at least 3 washes with PBST, the cells were incubated with fluorescent secondary antibodies for 1 h at room temperature in the dark. The slides were mounted against a coverslip using fluorescence antiquenching mountant Fluoromount G containing DAPI (Invitrogen, 00-4958-02) and sealed with nailpolish. The images were acquired using a laser confocal microscope LSM 710 (Zeiss) and analyzed with Zen software (Zeiss).

To quantify the pUb intensity in U2OS cells, confocal images were subjected to intensity analysis using FiJi and a macro file produced in-house, as previously reported [30].

### 4.10. Super Resolution Imaging by Structure Illuminated Matrix (SIM)

Fixed cells stained with pUb antibody, γ-Tubulin and DAPI were imaged using structure illuminated matrix (SIM) on a Zeiss Lattice SIM (Andor1, AxioObserver) using a Plan-Apochromat X63/NA 1.46 oil immersion lens. Four lasers were used for the different tags: UV diode laser 405 nm (DAPI), Blue diode laser 488 nm (Alexa Fluor 488), Green diode laser 561 nm (Alexa Fluor 555), and Red diode laser 642 nm (Cy5, AF647). Images were processed in ZEN black 3.0 by SIM algorithm using default parameters (Low SNR Input, 16 iterations).

### 4.11. Cell Cycle Analysis Using Flow Cytometry

Freshly harvested U2OS cells (by either trypsinization or mitotic shake-off) were washed thrice with PBS. Cells were then suspended in ice-cold PBS to a single cell suspension and ice-cold EtOH was added to a final 70% (*v*/*v*). The cells were kept in −20 °C for a minimum of 12 h and then washed thrice with cold PBS. The cells were then permeabilized by incubating for 5 min with PBS containing 1% TRITON X-100 at room temperature. Non-specific interactions were blocked for 1 h with 0.5% BSA in PBS containing 0.02% NaN3 at room temperature. Subsequently, Alexa fluor 488 conjugated pUb antibody (1:500 dilution) in a stabilizing solution (Candor, 130500) was directly added to the cells and kept for 1 h at room temperature. Cell were washed with PBS containing 0.05% of Tween 20 (PBST) to remove the excess antibody and suspended in FxCycleTM PI/RNase Staining Solution (Invitrogen, F10797). The stained cells were analyzed using Cytek Aurora spectral flow cytometer. The cell cycle and pUb subpopulations were analyzed using FCS Express software (Pasadena, CA, USA).

### 4.12. Generation of PINK1 Knockout Cells

The following sequence was used to design the guide RNA for targeting the PINK1 sequence: 5′-TCGACCCAGCTGCAGGCCG-3′. The self-complementary guide RNA oligonucleotides were annealed and their 5′ ends were phosphorylated using T4 polynucleotide kinase. The annealed guide RNA was ligated into the BpiI digested pSp-Cas9-BB-2A-Puro PX459 V 2.0 plasmid (Addgene plasmid#62988). The positive transformants were screened by colony PCR, restriction digestion and sequencing. The PINK1 CRISPR plasmid was transfected into U2OS cells and 48 h post transfection the cells were selected with 2 µg/mL Puromycin until isolated colonies were observed. The colonies were expanded and screened individually for PINK1 expression by Western blotting followed by confirming the intended microdeletion by DNA sequencing.

### 4.13. Site-Directed Mutagenesis in Ubiquitin S65A, S65E

pcDNA3 HA-Ubiquitin plasmid was obtained from Addgene (Plasmid#18712). To mutate S65 in Ubiquitin to Alanine following primers were designed:

FW: 5′-CAACATCCAGAAGGAGGCGACCCTGCACCTGG-3′;

RV: 5′-CCAGGTGCAGGGTCGCCTCCTTCTGGATGTTG-3′.

In order to mutate S65 to Glutamic acid following primers were used:

FW: 5′-CAACATCCAGAAGGAGGAGACCCTGCACCTGG-3′;

RV: 5′- CCAGGTGCAGGGTCTCCTCCTTCTGGATGTTG-3′.

In total, 100 ng of the plasmid DNA was PCR amplified with a pair of primers with the intended mutation. The amplified DNA was digested with the DpnI restriction enzyme overnight to eliminate the methylated parental DNA. The DpnI digested DNA was transformed into DH5α and the transformants were randomly screened for the presence of the intended mutation in the ubiquitin sequence by DNA sequencing.

## 5. Phosphoproteomics

### 5.1. Proteolysis

The cell pellets were dissolved in in Lysis buffer (8M urea, 75mM NaCl, 50 mM Tris pH = 8.2, Protease inhibitor, 1mM NaF, 1 mM β-glycerophosphate, 1 mM Sodium Orthovandate, 10 mM Sodium Pyrophosphate, 1 mM PMSF), sonicated three times (90%, 10–10 cycles, 5’), and centrifuged. A total of 1.5 mg protein from each sample were reduced (60 °C for 30 min), modified with 35.2 mM mM iodoacetamide in 400 mM ammonium bicarbonate (in the dark, room temperature, for 30 min) and digested in 1.5 M Urea, 67 mM ammonium bicarbonate with modified trypsin (Promega) at a 1:50 enzyme-to-substrate ratio, overnight, at 37 °C. An additional second trypsinization was done for 4 h. The proteins in the solution were reduced (60 °C for 30 min), modified with 35.2 mM iodoacetamide in 100 mM ammonium bicarbonate (in the dark, room temperature, for 30 min) and digested in 1.5 M Urea, 70 mM ABC with modified trypsin (Promega) at a 1:50 enzyme-to-substrate ratio, overnight, at 37 °C. A second digestion was done for 4 h.

### 5.2. Dimethyl Labeling and Phosphopeptides Enrichment

The resulting tryptic peptides were desalted using C18 tips (Oasis, Waters), dried and re-suspended in 50 mM Hepes (pH 7.9). Labeling by Dimethylation was done in the presence of 100 mM NaCBH3 (Sterogene cat#9704 1M), by adding light formaldehyde (35% Frutarom cat#5551810, 12.3 M) to one of the samples, and heavy formaldehyde (20% *w*/*w*, Cambridge Isotope laboratories cat#CDLM-4599-1,6.5 M) to the other sample to a final concentration of 200 mM. The sample was incubated for 1 h. Neutralization was done with 1 M ABC for 30 min and equal amounts of the light and heavy peptides were mixed, cleaned on C18 TopTip columns and re-suspended in 40% Acetonitrile (ACN), 6% TFA (Trifluoroacetic acid), and enriched for phosphopeptides on titanium dioxide (TiO2) beads. A two sample pair labeled H and L were combined G1S(H) with G2M(L), G1S+CCCP(H) with G2M(L). Titanium beads were pre-washed (80% ACN, 6% TFA) mixed with the peptides for 10 min at 37 °C, washed with 30% ACN with 3% TFA, and 80% ACN with 0.1% TFA. Bound peptides were eluted with 20% ACN with 325 mM ammonium hydroxide followed by 80% ACN with 325 mM ammonium hydroxide. The resulted peptides were desalted using C18 tips and analyzed by LC-MS-MS.

### 5.3. Mass Spectrometry Analysis

The resulted peptides were analyzed by LC-MS/MS using a Q Exactive plus mass spectrometer (Thermo) fitted with a capillary HPLC (easy nLC 1000, Thermo-Fisher, Waltham, MA, USA). The peptides were loaded onto a C18 trap column (0.3 × 5 mm, LC-Packings) connected on-line to a homemade capillary column (20 cm, 75 micron ID) packed with Reprosil C18-Aqua (Dr. Maisch GmbH, Ammerbuch, Germany) in solvent A (0.1% formic acid in water). The peptides mixture was resolved with a (5 to 28%) linear gradient of solvent B (95% acetonitrile with 0.1% formic acid) for 180 min followed by gradient of 15 min gradient of 28 to 95% and 25 min at 95% acetonitrile with 0.1% formic acid in water at flow rates of 0.15 μL/min. Mass spectrometry was performed in a positive ion mode (at mass range of *m*/*z* 350–1800 AMU and resolution 70,000) using repetitively full MS scan followed by collision induced dissociation (HCD, at 35 normalized collision energy) of the 10 most dominant ions (>1 charges) selected from the first full MS scan.

### 5.4. Mass Spectrometry Data Analysis

The mass spectrometry data were analyzed using the MaxQuant software 1.5.2.8. (Munich, Germany) (www.maxquant.org, ref accessed on 29 April 2021) for peak picking identification and quantitation using the Andromeda search engine, searching against the human proteome of the Uniprot database with mass tolerance of 20 ppm for the precursor masses and 20 ppm for the fragment ions. Methionine oxidation, phosphorylation (STY) and protein N-terminus acetylation were accepted as variable modifications and carbamidomethyl on cysteine was accepted as static modifications. Minimal peptide length was set to six amino acids and a maximum of two miscleavages was allowed. Peptide- and protein-level false discovery rates (FDRs) were filtered to 1% using the target-decoy strategy. The protein table was filtered to eliminate the identifications from the reverse database, and common contaminants. The data were quantified by H/L ratio of the demethylated peptides using the MaxQuant software. Perseus software was used for statistical analysis of the data.

### 5.5. “In gel” Proteolysis and Mass Spectrometry Analysis of the IP Samples

The proteins in the gel were reduced with 3 mM DTT (60 °C for 30 min), modified with 10 mM iodoacetamide in 100 mM ammonium bicarbonate (in the dark, room temperature, for 30 min) and digested in 10% acetonitrile and 10 mM ammonium bicarbonate with modified trypsin (Promega) at a 1:10 enzyme-to-substrate ratio, overnight, at 37 °C. The resulted peptides were desalted using C18 tips (homemade stage tips) and were subjected to LC-MS-MS analysis. The peptides were resolved by reverse-phase chromatography on 0.075 × 180-mm fused silica capillaries (J&W) packed with Reprosil reversed phase material (Dr Maisch GmbH, Germany). They were eluted with linear 60 min gradient of 5 to 28% 15 min gradient of 28 to 95% and 15 min at 95% acetonitrile with 0.1% formic acid in water at flow rates of 0.15 μL/min. Mass spectrometry was performed by Q Exactive plus mass spectrometer (Thermo) in a positive mode using repetitively full MS scan followed by collision induced dissociation (HCD) of the 10 most dominant ions selected from the first MS scan.

### 5.6. Mass Spectrometry Data Analysis

The mass spectrometry data were analyzed using Proteome Discoverer 2.4 software with Sequest (Thermo) search engine against the human proteome from the Uniprot database with mass tolerance of 20 ppm for the precursor masses and 0.05 amu for the fragment ions. Oxidation on Met, Phosphorylation on Ser and Thr, Ubiquitination on Lys were accepted as variable modifications, carbamidomethyl on Cys was accepted as static modifications. Minimal peptide length was set to six amino acids and a maximum of two miscleavages was allowed. Peptide- and protein-level false discovery rates (FDRs) were filtered to 1% using the target-decoy strategy. The protein table was filtered to eliminate the identifications from common contaminants and single-peptide identifications. Semi quantitation was done by calculating the peak area of each peptide based on its extracted ion currents (XICs) and the area of the protein is the average of the sum of all the identified peptides from each protein. Pathway analysis was done using the string software (https://string-db.org/ accessed on 29 April 2021).

## Data Availability

The data presented in this study are available on request from the corresponding author.

## Supplementary Materials

The following supporting information can be downloaded at: https://www.mdpi.com/article/10.3390/molecules27154867/s1. Figure S1: The phospho-Ubiquitin (pUb) signal corresponds to the mitotic cell population.; Figure S2: The pUb signal is unremovable by the deubiquitinases.; Figure S3: PINK1 KO U2OS cells and parkin overexpression affect the pUb.; Figure S4: PINK1 is important for cell cycle progression through mitosis and thus cell proliferation.; Figure S5: Identifying the proteins conjugated with pUb.; Figure S6: CENPF is not covalently conjugated with pS65 Ub.; Figure S7: PLK1 kinase generates pUb signal during mitosis.; Figure S8: Wild type U2OS cells were transfected with plasmids expressing HA tagged wild type, S65A and S65E mutant Ubiquitin plasmids followed by their mitotic arrest by using Colcemid.; Figure S9: Cellular distribution of Synthetic Ub probe and pUb antibody in U2OS cells.; Figure S10: Mitotic entry effect on pUb peptide peak compared to CCCP treatment in U2OS cells with stable expression of untagged human parkin; Table S1: List of proteins that were significantly enriched.

## Sample Availability

Samples of the compounds tested are not available from the authors.
