# Supplementary material for: Antibody for Serine 65 Phosphorylated Ubiquitin Identifies PLK1-Mediated Phosphorylation of Mitotic Proteins and APC1
Source: Molecules. 2022 Jul 29;27(15):4867. doi: 10.3390/molecules27154867 (PMC9369648; doi:10.3390/molecules27154867)
Supplement: Supplementary file 1 [file molecules-27-04867-s001.zip › molecules-1807520-supplementary.pdf]

Supplementary Material

Antibody for Serine 65 Phosphorylated Ubiquitin Identifies PLK1-Mediated Phosphorylation of Mitotic Proteins and APC1

Guy Mann [1,†], Prasad Sulkshane [2,†], Pradeep Sadhu [1], Tamar Ziv [3], Michael H. Glickman [2,*] and Ashraf Brik [1,*]

[1] Schulich faculty of Chemistry, Technion–Israel Institute of Technology, Haifa 32000, Israel; guym@campus.technion.ac.il (G.M.); pradeepsadhu@campus.technion.ac.il (P.S.)
[2] Faculty of Biology, Technion–Israel Institute of Technology, Haifa 32000, Israel; prasads@campus.technion.ac.il
[3] The Smoler Protein Research Center, Lorry I. Lokey Interdisciplinary Center for Life Sciences and Engineering, Technion Israel Institute of Technology, Haifa 32000, Israel; tamarz@technion.ac.il
† These authors contributed equally to this work
* Correspondence: abrik@technion.ac.il (A.B.); glickman@technion.ac.il (M.H.G.)

Contents

List of antibodies used in the study 2

Supplementary figures 2

Supplementary figure S1 .... **Error! Bookmark not defined.**

Supplementary figure S2 .... **Error! Bookmark not defined.**

Supplementary figure S3 .... **Error! Bookmark not defined.**

Supplementary figure S4 .... **Error! Bookmark not defined.**

Supplementary figure S5 .... **Error! Bookmark not defined.**

Supplementary figure S6 .... **Error! Bookmark not defined.**

Supplementary figure S7 .... **Error! Bookmark not defined.**

Supplementary figure S8 .... **Error! Bookmark not defined.**

Supplementary figure S9 .... **Error! Bookmark not defined.**

Supplementary figure S10: .... **Error! Bookmark not defined.**

Supplementary tables 9

Supplementary Table 1 .... **Error! Bookmark not defined.**

1. List of antibodies used in the study.

Antibody	Host species	Catalog number	Manufacturer
Phospho-ubiquitin (pS65)	Rabbit	ABS1513-I	Merck-Millipore
Phospho-ubiquitin (pS65)	Mouse	70973	Cell Signaling
Total Ubiquitin	Mouse	sc-8017	Santacruz Biotechnology
Total Ubiquitin (FK2)	Rabbit	04-263	Merck-Millipore
Gamma Tubulin	Mouse	MA5-31482	Invitrogen
Alpha Tubulin	Mouse	sc-5286	Santacruz Biotechnology
TOM20	Mouse	sc-17764	Santacruz Biotechnology
PINK1	Rabbit	6946	Cell Signaling
Parkin	Mouse	4211	Cell Signaling
PLK1	Rabbit	ABE2619	Merck-Millipore
Actin	Mouse	sc-81178	Santacruz Biotechnology
APC1	Rabbit	ab133397	Abcam
HA	Mouse	sc-7392	Santacruz Biotechnology
GAPDH	Rabbit	G9545	Sigma
BAZ2A	Rabbit	HPA063806	Sigma
SNRNP200	Rabbit	HPA029321	Sigma
RIF1	Rabbit	95558	Cell Signaling
CENP-F	Rabbit	ab264215	abcam
53BP1	Rabbit	ab175933	abcam

Supplementary figures

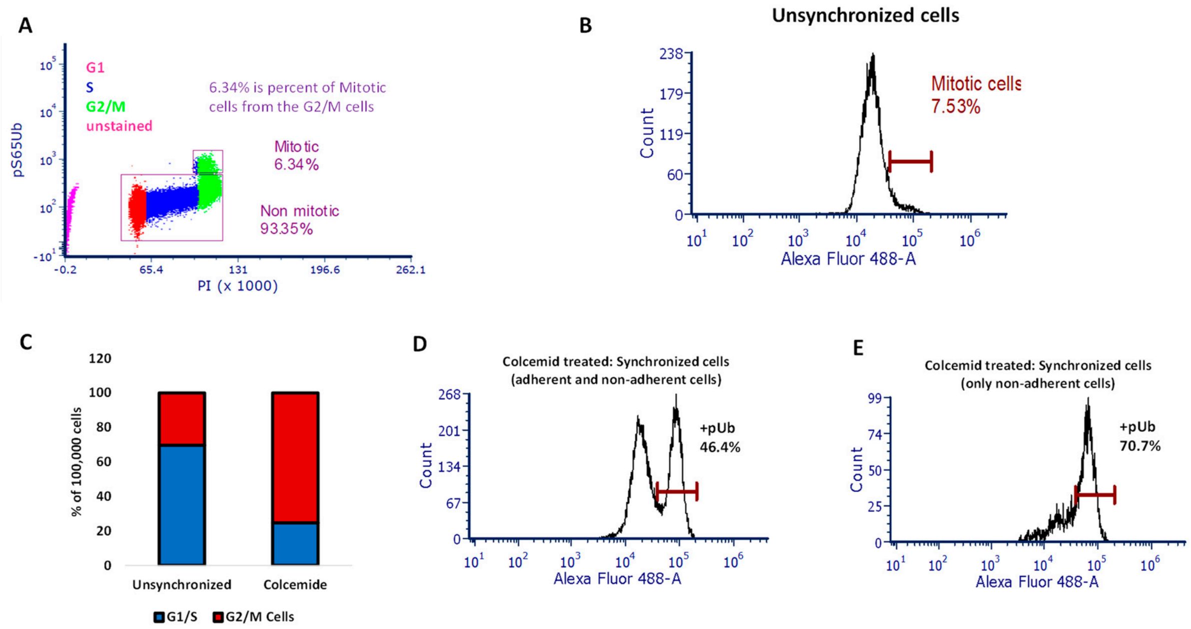

Figure S1. The phospho-Ubiquitin (pUb) signal corresponds to the mitotic cell population. **A**. Unsynchronized U2OS cells were stained with propidium iodide (PI) and Alexa 488-conjugated pS65 Ub antibody. The cell population was divided into G1, S and G2/M phases. The majority of the cell population (~93%) is negative for pUb staining and is supposed to be non-mitotic. A minor fraction of the cell population (~6%) shows pUb staining and is considered mitotic population. The data is representative of three independent experiments. **B.** A population of unsynchronized U2OS cells was analyzed for the expression of pUb, which correspond to ~7.5% cells positive for pUb staining and represent the mitotic population **C.** A population of unsynchronized and synchronized (Colcemid treated) U2OS cells were subjected to cell cycle analysis and represented as percent G1/S cells (blue) as opposed to percent G2/M cells (red). **D.** A synchronized (Colcemid-treated) population of U2OS cells comprising of the adherent as well as non-adherent population revealed ~46% cells positive for

pUb staining. **E.** A synchronized (Colcemid-treated) population of U2OS cells comprising only the adherent cells exhibited ~70% population positive for pUb.

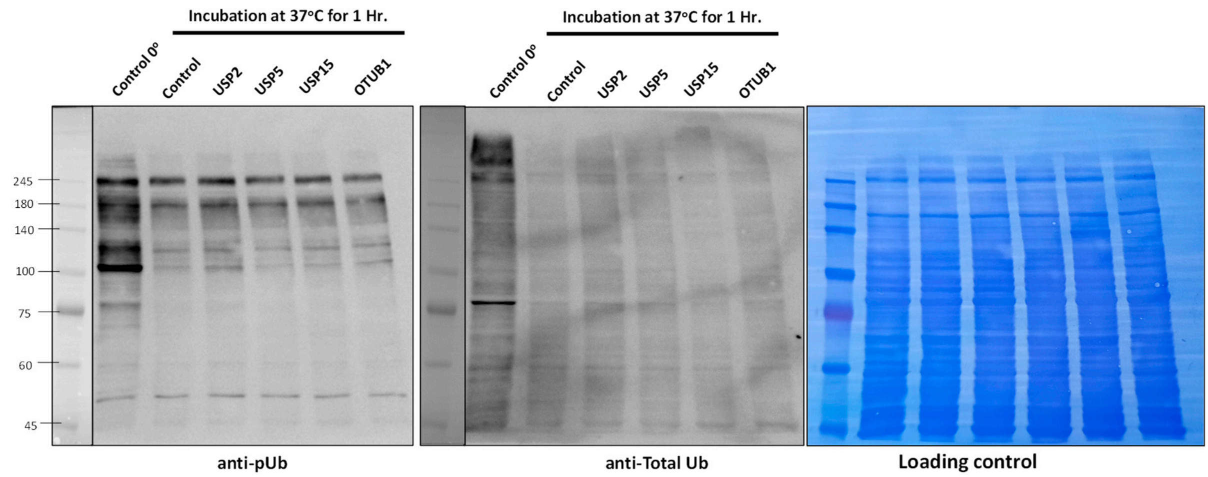

Figure S2. The pUb signal is unremovable by the deubiquitinases**.** Total cell lysate from mitotic U2OS cells was incubated with the indicated deubiquitinases for 1 hour at 37 C. The cell lysates were then subjected to immunoblotting with pUb and total Ub antibodies. The blots are representatives of three independent experiments.

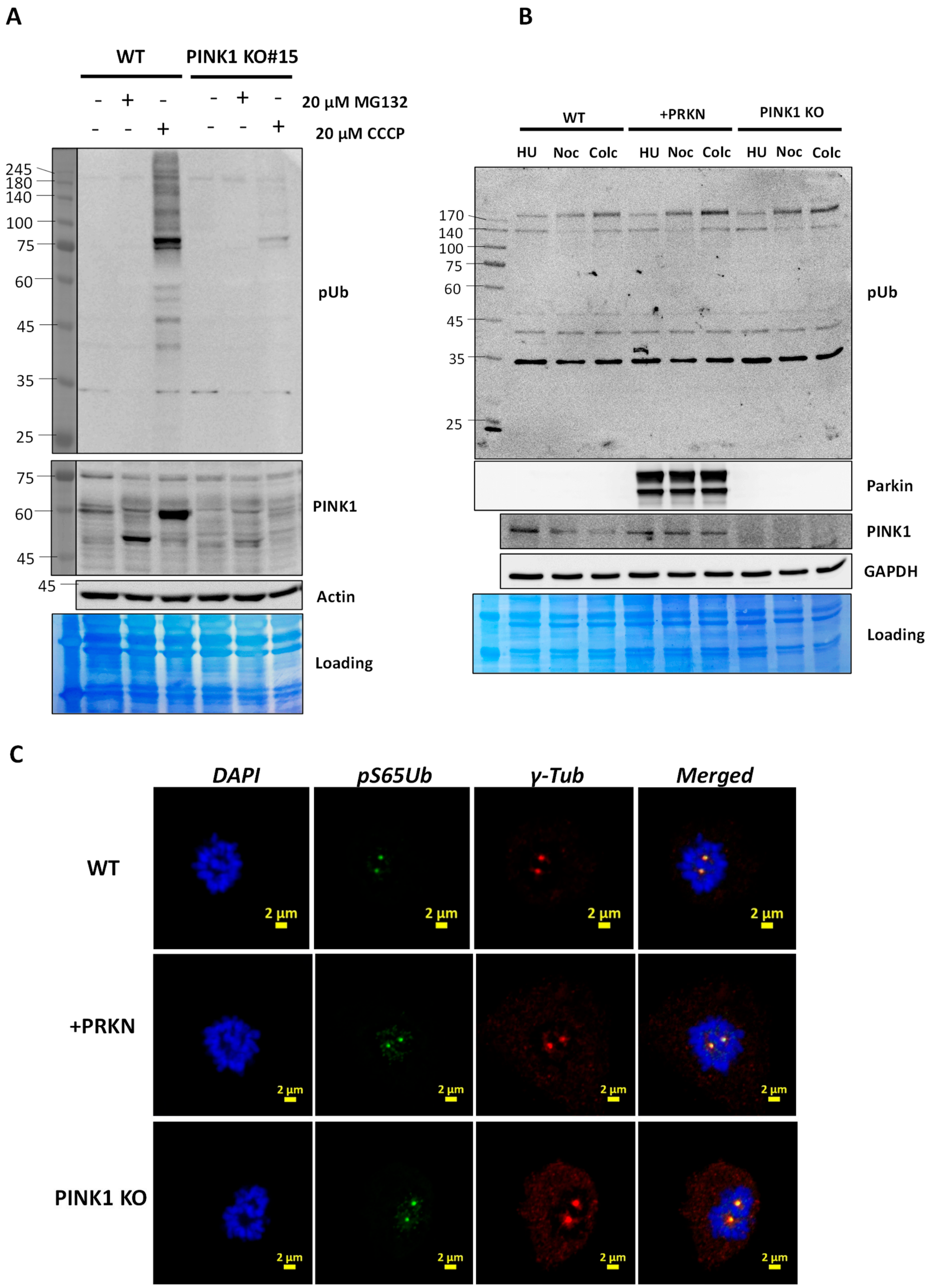

Figure S3. A. PINK1 kinase does not account for the pUb signal in mitotic cells. PINK1 KO U2OS cells were generated by CRISPR-Cas 9 approach and validated by PINK1 and pUb immunoblotting. The wild type cells exhibited stabilization of proteolytically cleaved form of PINK1 at about 50 Kda and CCCP treatment stabilized the full-length form of PINK1 which correlated with pUb smear. **B.** Parkin overexpression does not affect the pUb signal during mitosis. Wild type, Parkin overexpressing and PINK1 knockout U2OS cells were treated with Hydroxyurea (HU), Nocodazole (Noc) and Colcemid (Colc) and the whole cell extracts (adherent and non-adherent cells pooled together in case of Noc and Colc) were analyzed by immunoblotting using pUb antibody. Blots are representative of two independent experiments. **C.** Wild type, Parkin overexpressing and PINK1 knockout U2OS cells were arrested in mitosis by using Colcemid. The cells were fixed and

stained with Alexa 488-conjugated pUb-specific antibody, Alexa 647-conjugated γ-Tubulin antibody and DAPI. Images were acquired under a fluorescence confocal microscope.

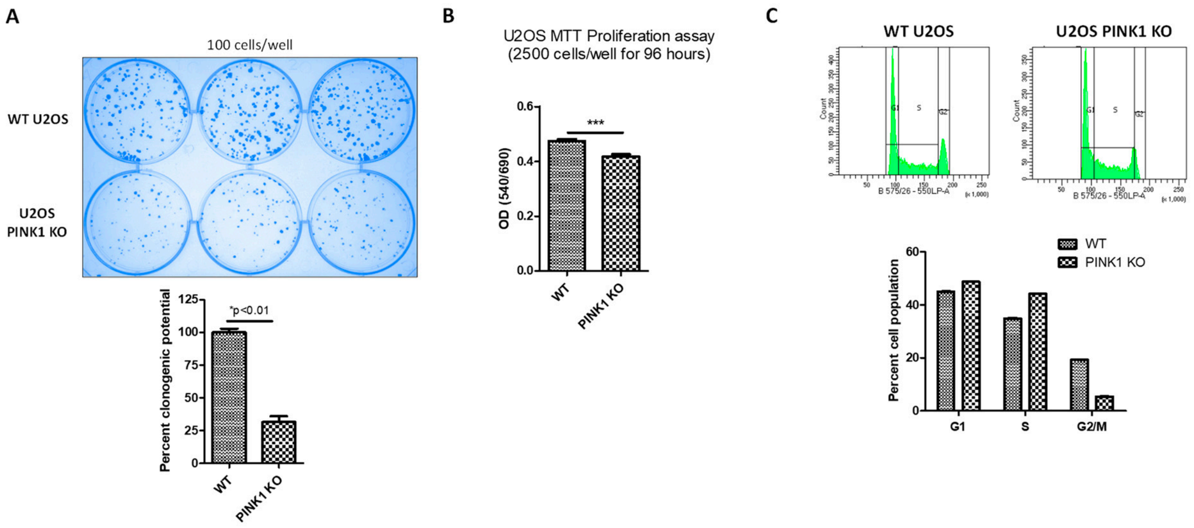

Figure S4. PINK1 is important for cell cycle progression through mitosis and thus cell proliferation. A. Clonogenic assay. 100 cells per well of both WT and PINK1 KO U2OS cells were seeded in triplicates. The cells were allowed to grow for about 2 weeks until visible colonies appear, which were then fixed, stained and counted manually. The percent clonogenic potential is represented as mean +/- SEM. **B.** MTT assay. WT and PINK1 KO cells were seeded (2500 cells per well) in 96 well plate in 10 replicates. The cells were allowed to grow for 96 hours and then followed by MTT assay. The cell viability is expressed in terms of OD at 540 nm with reference to 690 nm. **C.** Cell cycle analysis. WT and PINK1 KO U2OS cells were subjected to cell cycle analysis and the percentage of cells in the indicated cell cycle phases (G1, S, G2/M) were compared between the WT and PINK1 KO cells. PINK1 KO cells showed arrest in the G1 and S phases and lesser progression through the G2/M phase of the cell cycle. .

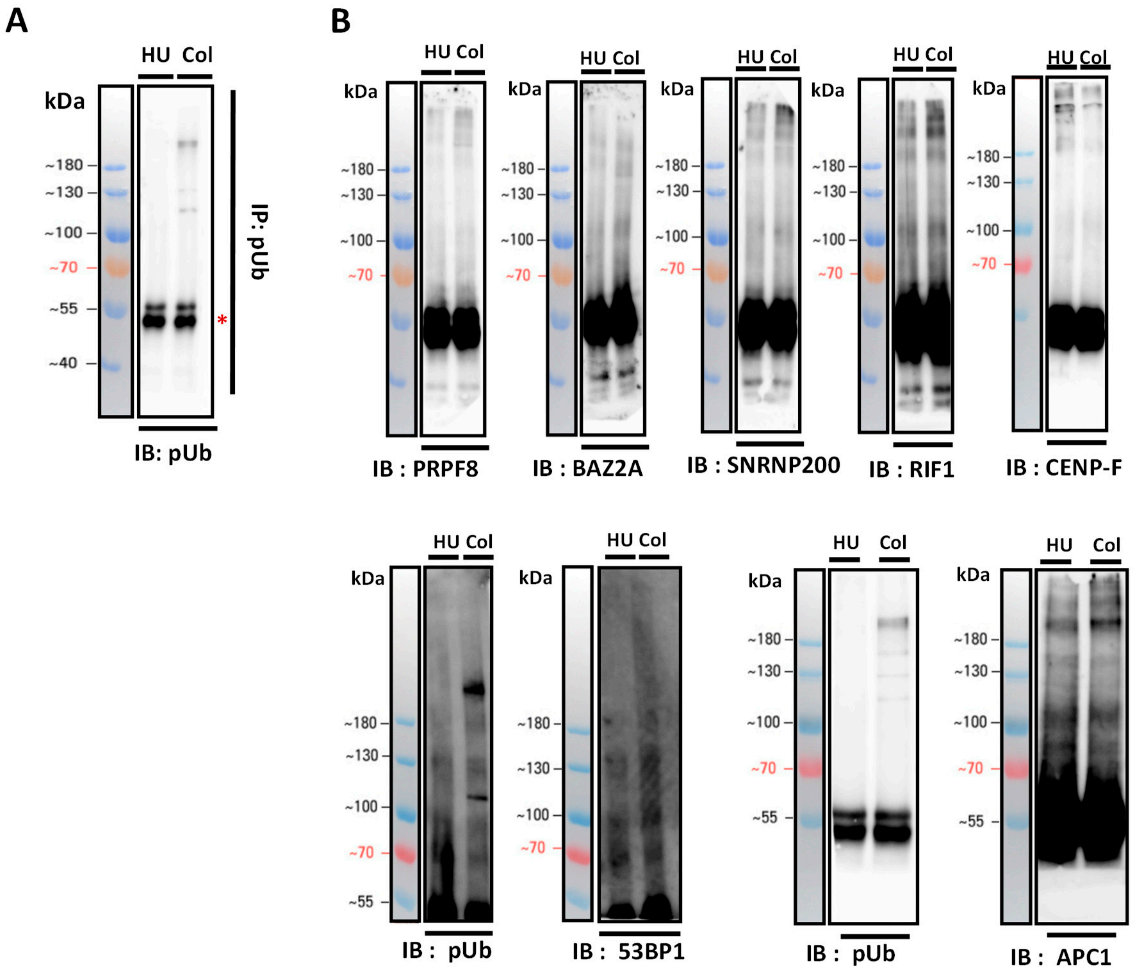

Figure S5. Identifying the proteins conjugated with pUb. **A.** Whole cell extracts from wild type U2OS cells treated with hydroxyurea (HU) or Colcemid (Col) were subjected to immunoprecipitation with pUb antibody followed by western blot analysis using the same antibody. Asterisk represents the antibody chain fragments. **B.** The IP fractions were probed with the indicated antibodies in addition to the pUb antibody.

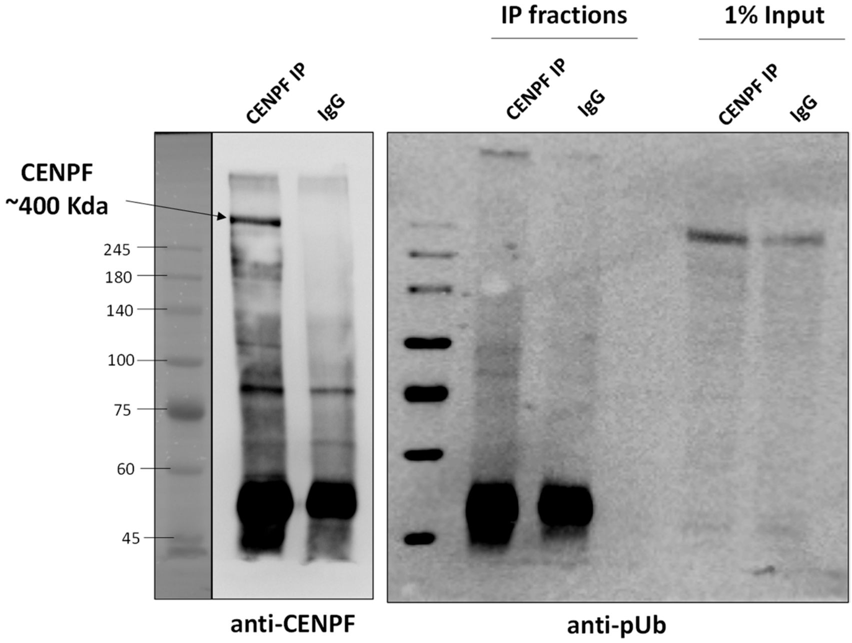

Figure S6. CENPF is not covalently conjugated with pS65 Ub. Colcemid treated mitotic lysates of wild type U2OS cells were subjected to immunoprecipitation with CENPF antibody and an isotype control (IgG) antibody. The IP fractions and 1% total input fractions were subjected to immunoblotting with antibodies specific for CENPF and pUb. pUb band was undetected at the same position as the CENPF, suggesting that CENPF was not covalently conjugated with pUb. .

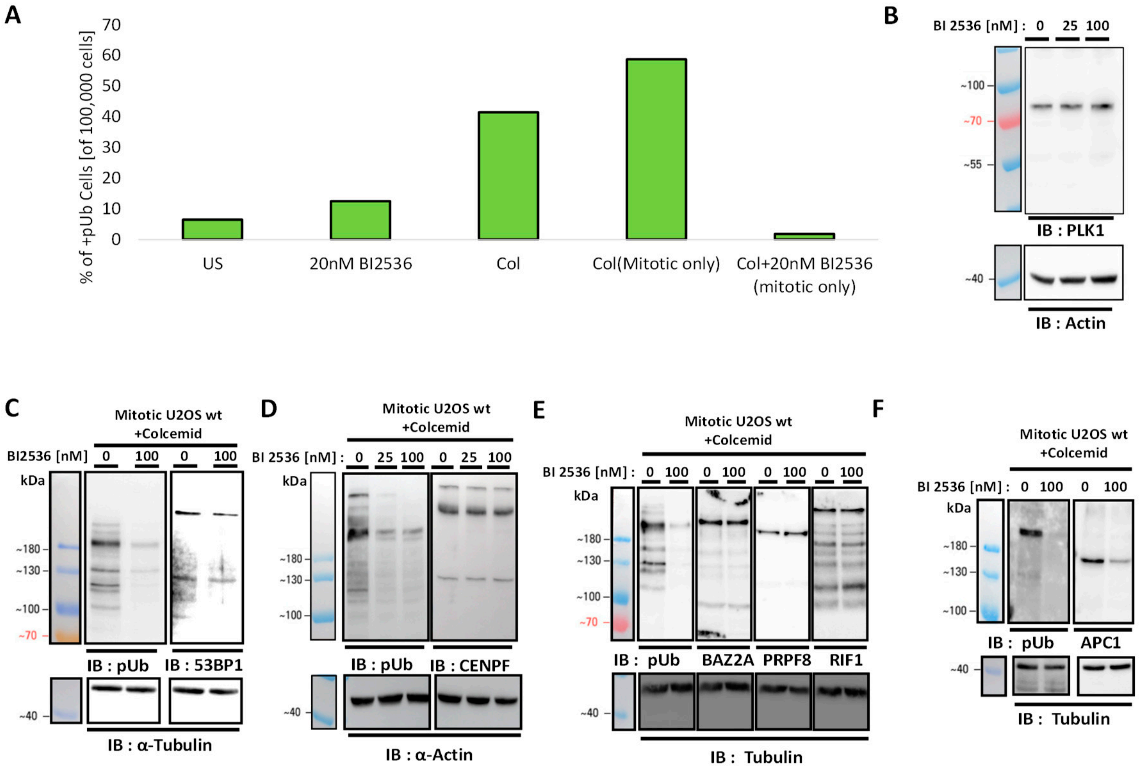

Figure S7. PLK1 kinase generates pUb signal during mitosis. **A.** PLK1 inhibition reduces the percent of pUb positive cells in flow cytometry (total 100,000 cells for each condition). US cells were harvested by trypsinization and mitotic cells were harvested by mitotic shake-off prior to fixation and staining with anti-pUb AF488 conjugated antibody. **B.** PLK1 inhibitor BI2536 does not alter PLK1 protein levels. Western blot analysis of whole cell extracts from wild type mitotic U2OS cells treated with indicated concentrations of BI2536 and immunoblotted for PLK1 and actin as a loading control. **C.** Mitotic wild type U2OS cells were treated with the indicated concentrations of BI2536 and the whole cell lysates were subjected to immunoblot analysis with pUb antibody and compared to anti-53BP1. **D.** pUb antibody compared to anti-CENPF. **E.** pUb antibody compared to anti-BAZ2A, anti-PRPF8 and anti RIF1. **F.** pUb antibody compared to anti-APC1.

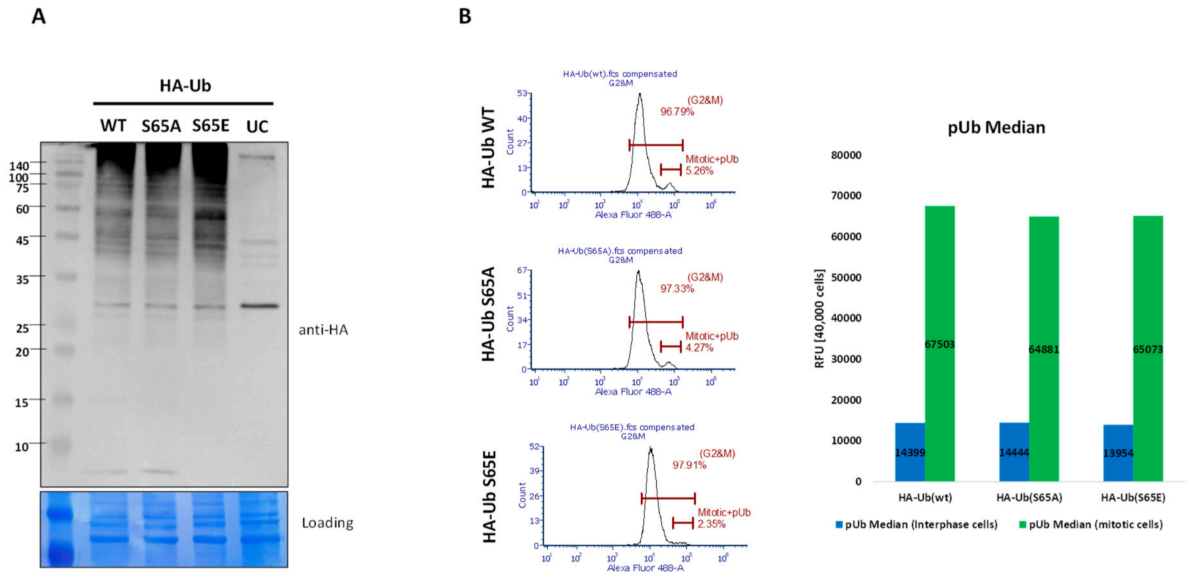

Figure S8. Wild type U2OS cells were transfected with plasmids expressing HA tagged wild type, S65A and S65E mutant Ubiquitin plasmids followed by their mitotic arrest by using Colcemid. **A.** The whole cell extracts were immunoblotted with HA antibody. "UC" indicate untransfected cells. **B.** Ectopic expression of mutant ubiquitin does not significantly diminish the mitotic pUb signal intensity and the mitotic population as determined by flow cytometry.

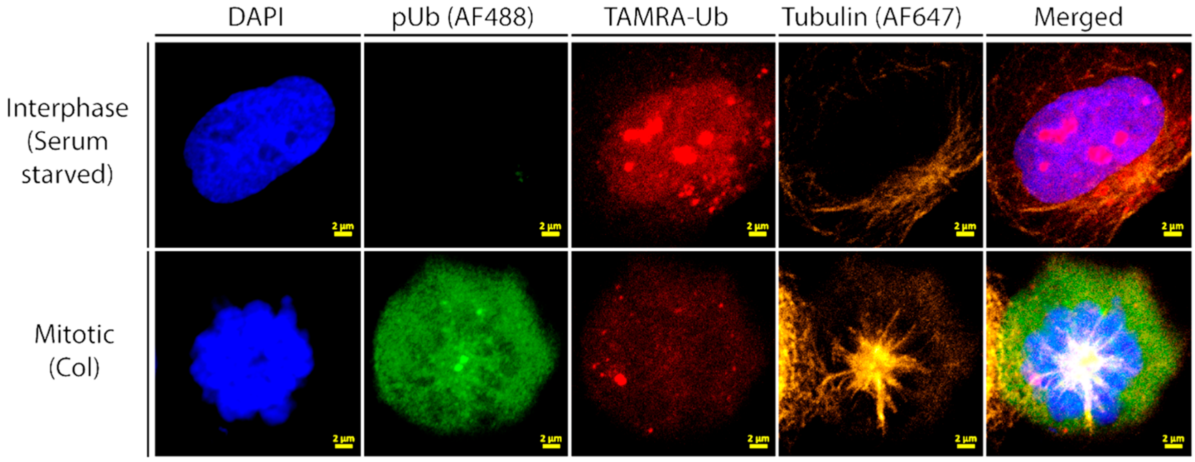

Figure S9. Cellular distribution of Synthetic Ub probe and pUb antibody in U2OS cells are different. LSCM images of TAMRA-Ub probe (Red), α-Tubulin (Gold), DAPI (Blue) and pUb (Green) in cells synchronized to interphase by serum starvation for 48 hours and mitosis by Colcemid treatment.

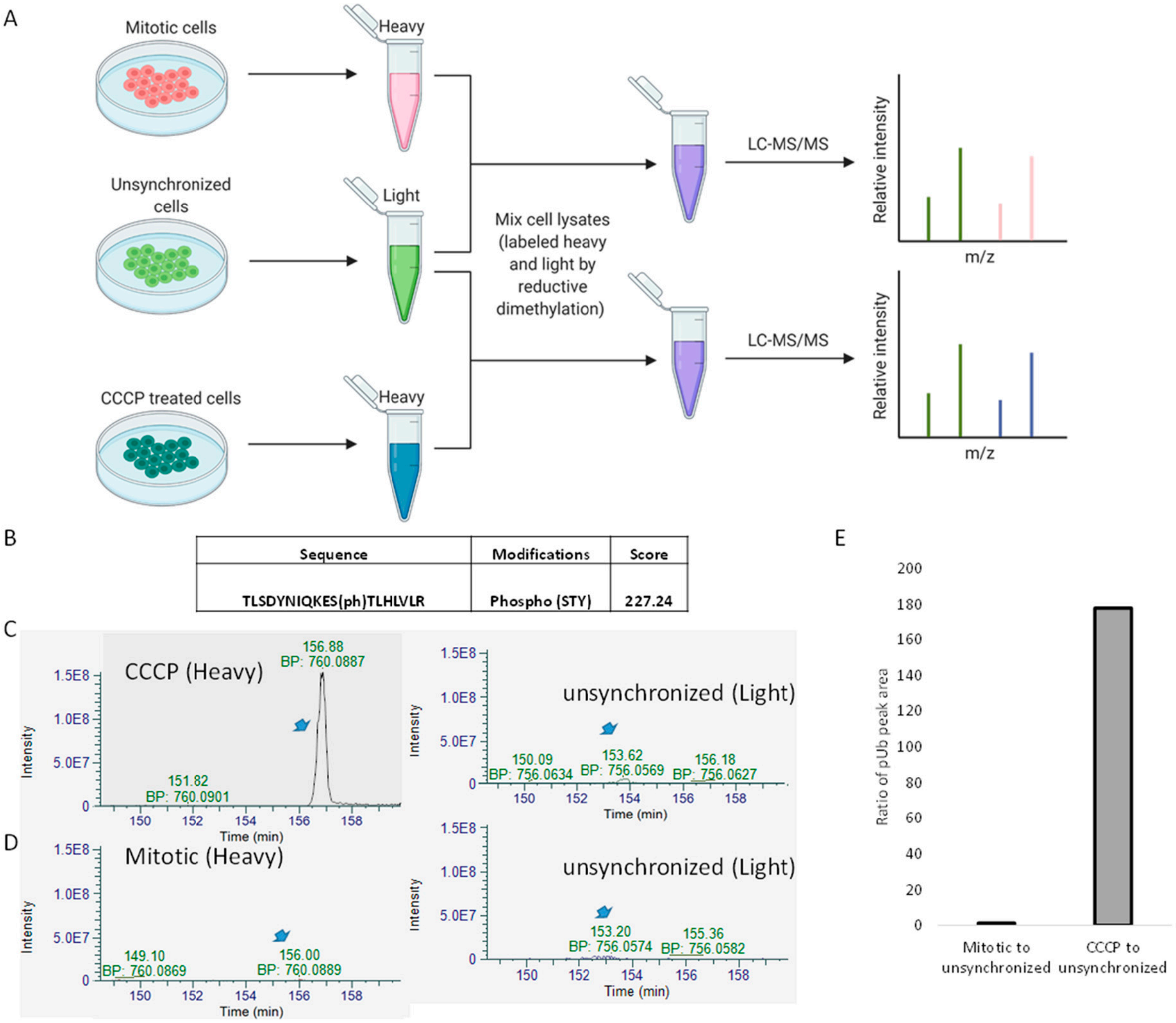

Figure S10. Mitotic entry effect on pUb peptide peak compared to CCCP treatment in U2OS cells with stable expression of untagged human parkin. **A.** Proteins from mitotic, CCCP treated and unsynchronized U2OS cells were trypsinized, labeled with reductive dimethylation and analyzed using LC-MSMS on the Q exactive HFX MS. Samples were enriched for the phosphopeptides. **B.** pUb peptide sequence and the Andromeda search engine score as identified from the phosphoproteome MS data that was analyzed against the human database. **C.** Ion chromatogram of labeled pUb in unsynchronized cells with CCCP (heavy), without CCCP (light). **D.** LC-MS/MS of pUb in Mitotic cells (heavy), and unsynchronized cells (light). Blue arrows indicate the identified peptide peak. **E.** Ratios of pUb peak area in Mitotic and CCCP treated cells compared to unsynchronized cells (from C&D).

Supplementary tables

Table S1. List of proteins that were significantly enriched (>2 fold in three independent experiments) by pUb antibody from mitotic lysates. .

Accession	Description	Gene Symbol
Q9H6R7	WD repeat and coiled-coil-containing protein OS=Homo sapiens OX=9606 GN=WDCP PE=1 SV=1	WDCP
Q96NY9	Crossover junction endonuclease MUS81 OS=Homo sapiens OX=9606 GN=MUS81 PE=1 SV=3	MUS81
Q8N680	Zinc finger and BTB domain-containing protein 2 OS=Homo sapiens OX=9606 GN=ZBTB2 PE=1 SV=1	ZBTB2
Q9ULR0	Pre-mRNA-splicing factor ISY1 homolog OS=Homo sapiens OX=9606 GN=ISY1 PE=1 SV=3	ISY1
P53350	Serine/threonine-protein kinase PLK1 OS=Homo sapiens OX=9606 GN=PLK1 PE=1 SV=1	PLK1

Q8TF05	Serine/threonine-protein phosphatase 4 regulatory subunit 1 OS=Homo sapiens OX=9606 GN=PPP4R1 PE=1 SV=1	PPP4R1
Q5T9A4	Serine/threonine-protein phosphatase 4 regulatory subunit 1 OS=Homo sapiens OX=9606 GN=PPP4R1 PE=1 SV=1	ATAD3B
Q9UKX2	Myosin-2 OS=Homo sapiens OX=9606 GN=MYH2 PE=1 SV=1	MYH2
Q8TD16	Protein bicaudal D homolog 2 OS=Homo sapiens OX=9606 GN=BICD2 PE=1 SV=1	BICD2
F2Z2E2	Ras GTPase-activating-like protein IQGAP3 OS=Homo sapiens OX=9606 GN=IQGAP3 PE=1 SV=1	IQGAP3
Q12834	Cell division cycle protein 20 homolog OS=Homo sapiens OX=9606 GN=CDC20 PE=1 SV=2	CDC20
O60508	Pre-mRNA-processing factor 17 OS=Homo sapiens OX=9606 GN=CDC40 PE=1 SV=1	CDC40
P49756	RNA-binding protein 25 OS=Homo sapiens OX=9606 GN=RBM25 PE=1 SV=3	RBM25
Q9BZJ0	Crooked neck-like protein 1 OS=Homo sapiens OX=9606 GN=CRNKL1 PE=1 SV=4	CRNKL1
O75691	Small subunit processome component 20 homolog OS=Homo sapiens OX=9606 GN=UTP20 PE=1 SV=3	UTP20
Q9P0V3	SH3 domain-binding protein 4 OS=Homo sapiens OX=9606 GN=SH3BP4 PE=1 SV=1	SH3BP4
Q9HC35	Echinoderm microtubule-associated protein-like 4 OS=Homo sapiens OX=9606 GN=EML4 PE=1 SV=3	EML4
Q16594	Transcription initiation factor TFIID subunit 9 OS=Homo sapiens OX=9606 GN=TAF9 PE=1 SV=1	TAF9
Q8TDM6	Disks large homolog 5 OS=Homo sapiens OX=9606 GN=DLG5 PE=1 SV=4	DLG5
A0A3B3IRR6	UPF0488 protein C8orf33 OS=Homo sapiens OX=9606 GN=C8orf33 PE=1 SV=1	C8orf33
P67809	Y-box-binding protein 1 OS=Homo sapiens OX=9606 GN=YBX1 PE=1 SV=3	YBX1
G3V325	ATP5MF-PTCD1 readthrough OS=Homo sapiens OX=9606 GN=ATP5MF-PTCD1 PE=4 SV=1	ATP5MF-PTCD1
O95347	Structural maintenance of chromosomes protein 2 OS=Homo sapiens OX=9606 GN=SMC2 PE=1 SV=2	SMC2
A0A6Q8PG37	Kinesin-like protein OS=Homo sapiens OX=9606 GN=KIF2A PE=1 SV=1	KIF2A
Q99728	BRCA1-associated RING domain protein 1 OS=Homo sapiens OX=9606 GN=BARD1 PE=1 SV=2	BARD1
Q92900	Regulator of nonsense transcripts 1 OS=Homo sapiens OX=9606 GN=UPF1 PE=1 SV=2	UPF1
Q12788	Transducin beta-like protein 3 OS=Homo sapiens OX=9606 GN=TBL3 PE=1 SV=2	TBL3
Q9H2Y7	Zinc finger protein 106 OS=Homo sapiens OX=9606 GN=ZNF106 PE=1 SV=1	ZNF106
Q9NSC5	Homer protein homolog 3 OS=Homo sapiens OX=9606 GN=HOMER3 PE=1 SV=2	HOMER3
Q15542	Transcription initiation factor TFIID subunit 5 OS=Homo sapiens OX=9606 GN=TAF5 PE=1 SV=3	TAF5
P62308	Small nuclear ribonucleoprotein G OS=Homo sapiens OX=9606 GN=SNRPG PE=1 SV=1	SNRPG
Q9Y3B4	Splicing factor 3B subunit 6 OS=Homo sapiens OX=9606 GN=SF3B6 PE=1 SV=1	SF3B6
P30414	NK-tumor recognition protein OS=Homo sapiens OX=9606 GN=NKTR PE=1 SV=2	NKTR
P46821	Microtubule-associated protein 1B OS=Homo sapiens OX=9606 GN=MAP1B PE=1 SV=2	MAP1B
Q86W56	Poly(ADP-ribose) glycohydrolase OS=Homo sapiens OX=9606 GN=PARG PE=1 SV=1	PARG
Q9UJS0	Calcium-binding mitochondrial carrier protein Aralar2 OS=Homo sapiens OX=9606 GN=SLC25A13 PE=1 SV=2	SLC25A13
H0YHA7	60S ribosomal protein L18 (Fragment) OS=Homo sapiens OX=9606 GN=RPL18 PE=1 SV=1	RPL18
H0Y6G2	Calcium/calmodulin-dependent protein kinase type II subunit gamma OS=Homo sapiens OX=9606 GN=CAMK2G PE=1 SV=2	CAMK2G
Q13554	Calcium/calmodulin-dependent protein kinase type II subunit beta OS=Homo sapiens OX=9606 GN=CAMK2B PE=1 SV=3	CAMK2B
O75369	Filamin-B OS=Homo sapiens OX=9606 GN=FLNB PE=1 SV=2	FLNB
P54198	Protein HIRA OS=Homo sapiens OX=9606 GN=HIRA PE=1 SV=2	HIRA
Q08188	Protein-glutamine gamma-glutamyltransferase E OS=Homo sapiens OX=9606 GN=TGM3 PE=1 SV=4	TGM3
P14635	G2/mitotic-specific cyclin-B1 OS=Homo sapiens OX=9606 GN=CCNB1 PE=1 SV=1	CCNB1
O95714	E3 ubiquitin-protein ligase HERC2 OS=Homo sapiens OX=9606 GN=HERC2 PE=1 SV=2	HERC2

F5H5D3	Tubulin alpha chain OS=Homo sapiens OX=9606 GN=TUBA1C PE=1 SV=1	TUBA1C
E9PK00	UV radiation resistance-associated gene protein (Fragment) OS=Homo sapiens OX=9606 GN=UVRAG PE=1 SV=1	UVRAG
P09012	U1 small nuclear ribonucleoprotein A OS=Homo sapiens OX=9606 GN=SNRPA PE=1 SV=3	SNRPA
Q8TDY2	RB1-inducible coiled-coil protein 1 OS=Homo sapiens OX=9606 GN=RB1CC1 PE=1 SV=3	RB1CC1
Q7RTV0	PHD finger-like domain-containing protein 5A OS=Homo sapiens OX=9606 GN=PHF5A PE=1 SV=1	PHF5A
Q8WU40	Calcium/calmodulin-dependent protein kinase (Fragment) OS=Homo sapiens OX=9606 GN=CAMK2G PE=1 SV=2	CAMK2G
Q6WCQ1	Myosin phosphatase Rho-interacting protein OS=Homo sapiens OX=9606 GN=MPRIP PE=1 SV=3	MPRIP
Q6PKG0	La-related protein 1 OS=Homo sapiens OX=9606 GN=LARP1 PE=1 SV=2	LARP1
Q86X51	EZH inhibitory protein OS=Homo sapiens OX=9606 GN=EZHIP PE=1 SV=1	EZHIP
Q12906	Interleukin enhancer-binding factor 3 OS=Homo sapiens OX=9606 GN=ILF3 PE=1 SV=3	ILF3
F5H538	Mitogen-activated protein kinase kinase kinase 4 OS=Homo sapiens OX=9606 GN=MAP3K4 PE=1 SV=1	MAP3K4
Q9P0K7	Ankycorbin OS=Homo sapiens OX=9606 GN=RAI14 PE=1 SV=2	RAI14
P62424	60S ribosomal protein L7a OS=Homo sapiens OX=9606 GN=RPL7A PE=1 SV=2	RPL7A
Q6IPR3	tRNA wybutosine-synthesizing protein 3 homolog OS=Homo sapiens OX=9606 GN=TYW3 PE=1 SV=2	TYW3
P61964	WD repeat-containing protein 5 OS=Homo sapiens OX=9606 GN=WDR5 PE=1 SV=1	WDR5
M0R0R2	40S ribosomal protein S5 OS=Homo sapiens OX=9606 GN=RPS5 PE=1 SV=1	RPS5
P62269	40S ribosomal protein S18 OS=Homo sapiens OX=9606 GN=RPS18 PE=1 SV=3	RPS18
H0Y993	DEK oncogene (DNA binding), isoform CRA_b OS=Homo sapiens OX=9606 GN=DEK PE=1 SV=2	DEK
P21127	Cyclin-dependent kinase 11B OS=Homo sapiens OX=9606 GN=CDK11B PE=1 SV=4	CDK11B
P21333	Filamin-A OS=Homo sapiens OX=9606 GN=FLNA PE=1 SV=4	FLNA
K7EQJ5	40S ribosomal protein S15 OS=Homo sapiens OX=9606 GN=RPS15 PE=1 SV=2	RPS15
P11940	Polyadenylate-binding protein 1 OS=Homo sapiens OX=9606 GN=PABPC1 PE=1 SV=2	PABPC1
P36578	60S ribosomal protein L4 OS=Homo sapiens OX=9606 GN=RPL4 PE=1 SV=5	RPL4
A0A087X0X3	Heterogeneous nuclear ribonucleoprotein M OS=Homo sapiens OX=9606 GN=HNRNPM PE=1 SV=1	HNRNPM
Q9H6E5	Speckle targeted PIP5K1A-regulated poly(A) polymerase OS=Homo sapiens OX=9606 GN=TUT1 PE=1 SV=2	TUT1
P19474	E3 ubiquitin-protein ligase TRIM21 OS=Homo sapiens OX=9606 GN=TRIM21 PE=1 SV=1	TRIM21
P62917	60S ribosomal protein L8 OS=Homo sapiens OX=9606 GN=RPL8 PE=1 SV=2	RPL8
P49848	Transcription initiation factor TFIID subunit 6 OS=Homo sapiens OX=9606 GN=TAF6 PE=1 SV=1	TAF6
Q96RL1	BRCA1-A complex subunit RAP80 OS=Homo sapiens OX=9606 GN=UIMC1 PE=1 SV=2	UIMC1
B1ALK7	Rho guanine nucleotide exchange factor 7 OS=Homo sapiens OX=9606 GN=ARHGEF7 PE=1 SV=1	ARHGEF7
Q9BX63	Fanconi anemia group J protein OS=Homo sapiens OX=9606 GN=BRIP1 PE=1 SV=2	BRIP1
Q86YT6	E3 ubiquitin-protein ligase MIB1 OS=Homo sapiens OX=9606 GN=MIB1 PE=1 SV=1	MIB1
A0A2R8YFX5	Neuron navigator 3 OS=Homo sapiens OX=9606 GN=NAV3 PE=1 SV=1	NAV3
Q15149	Plectin OS=Homo sapiens OX=9606 GN=PLEC PE=1 SV=3	PLEC
P13807	Glycogen [starch] synthase, muscle OS=Homo sapiens OX=9606 GN=GYS1 PE=1 SV=2	GYS1
Q13557	Calcium/calmodulin-dependent protein kinase type II subunit delta OS=Homo sapiens OX=9606 GN=CAMK2D PE=1 SV=3	CAMK2D
Q2M1P5	Kinesin-like protein KIF7 OS=Homo sapiens OX=9606 GN=KIF7 PE=1 SV=2	KIF7
P68371	Tubulin beta-4B chain OS=Homo sapiens OX=9606 GN=TUBB4B PE=1 SV=1	TUBB4B
O43172	U4/U6 small nuclear ribonucleoprotein Prp4 OS=Homo sapiens OX=9606 GN=PRPF4 PE=1 SV=2	PRPF4
